# Crystal structure of 5-[2-(2,4,6-tri­bromo­phen­yl)diazen­yl]tropolone

**DOI:** 10.1107/S2056989018006151

**Published:** 2018-04-27

**Authors:** Tania N. Hill, Kelsey L. Savig, Andreas Lemmerer

**Affiliations:** a Molecular Sciences Institute, School of Chemistry, University of the Witwatersrand, PO WITS 2050, Johannesburg, South Africa

**Keywords:** crystal structure, azotropolone, azo group

## Abstract

The title compound {systematic name: 2-hy­droxy-5-[2-(2,4,6-tri­bromo­phen­yl)diazen-1-yl]cyclo­hepta-2,4,6-trien-1-one}, C_13_H_7_Br_3_N_2_O_2_, is essentially planar, with an r.m.s. deviation of 0.054 Å. The mol­ecular structure is fixed in the azo tautomer by intra­molecular C—H⋯N inter­actions, with O—H⋯O hydrogen bonds creating linked dimers. Charge-transfer inter­actions are observed, with the segregated stacks linked by Br⋯Br inter­actions.

## Abstract 2

The title compound is essentially planar, with an r.m.s. deviation of 0.054 Å. The mol­ecular structure is fixed in the azo tautomer by intra­molecular C—H⋯N inter­actions, with O—H⋯O hydrogen bonds creating linked dimers. Charge-transfer inter­actions are observed, with the segregated stacks linked by Br⋯Br inter­actions.

## Chemical context   

**Figure 1:**
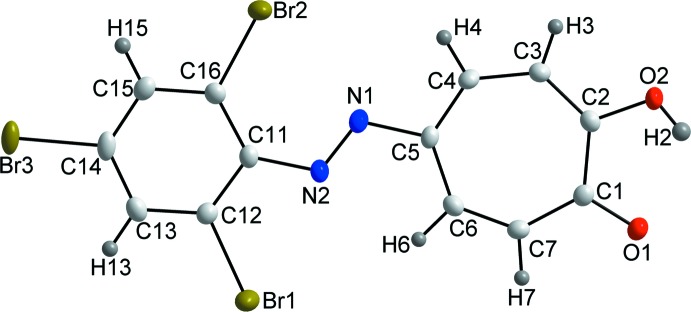
View of **I** with 50% probability displacement ellipsoids

In modern times, dyes have become an enormous market (approx. $13.4 billion) with over one million tons of various dyes and pigments being produced each year, and their uses ranging from textiles, cosmetics, food coloring, printing inks to optical recording media. Azo dyes form one of the largest groups of synthetic chemical dyes with the chemical structure of these compounds featuring substituted aromatic rings that are joined by one or more azo groups (*R*—N=N—*R*). The first of these dyes to be produced was aniline yellow, which contains an azo-substituted benzene group. Tropolone exhibits similar aromatic properties to benzene; as such, a study was undertaken to synthesize a series of azo-functionalized tropolones. A search of the Cambridge Structural Database (CSD; Groom *et al.*, 2016 ▸) yielded twenty troponoid compounds with a mono-substituted 5-position. Of these, only eleven had the tropolone backbone, only two of which had an azo linking group. As part of this study, we report the crystal structure of the title compound, **I** (Fig. 1 ▸).
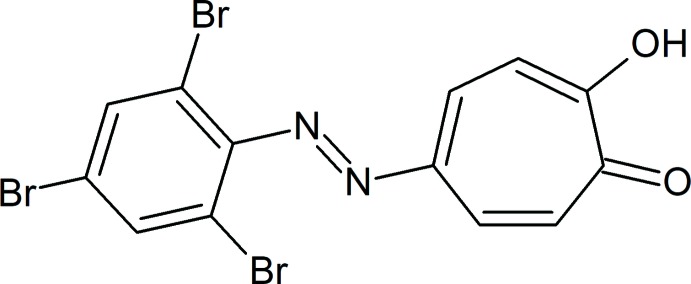


## Structural commentary   

**Figure 2:**
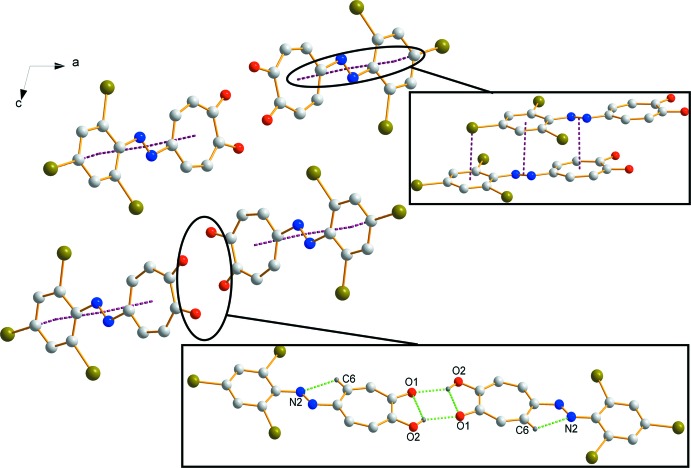
The packing of **I** as viewed along the *b*-axis. The top-right insert illustrates the Br⋯π and π→π^*^ CT inter­actions, while the bottom-right insert illustrates the O—H⋯O and C—H⋯N inter­actions as dashed bonds. Only selected hydrogen atoms are shown for clarity.

The title compound, **I**, shows no signs of azo-hydrazone tautomerization, a phenomenon known to the phenyl­azo-derivatives, because of the stabilizing intra­molecular inter­action of the hydrogen (H6) atom of the tropolone ring with the nitro­gen (N2) atom of the azo group (Table 1 ▸, Fig. 2 ▸). Similar to 5-phenyl­azotropolone (Hill *et al.*, 2012 ▸), **I** is essentially planar, with the dihedral angle between the least-squares planes *A* (O1, O2, C1–C7, N1) and *B* (N2, C11–C16) of 5.07 (6)° with an r.m.s. deviation of 0.054 Å. The largest variation from the mol­ecular plane is for the phenyl carbon (C13) with a value of 0.096 (2) Å. However, this planarity does not extend to the other azotropolones: 5-(4-eth­oxy­phenyl­azo)tropolone (Kubo *et al.*, 2008 ▸) was found to be twisted with an angle of 27.6 (1)°.

## Supra­molecular features   

**Figure 3:**
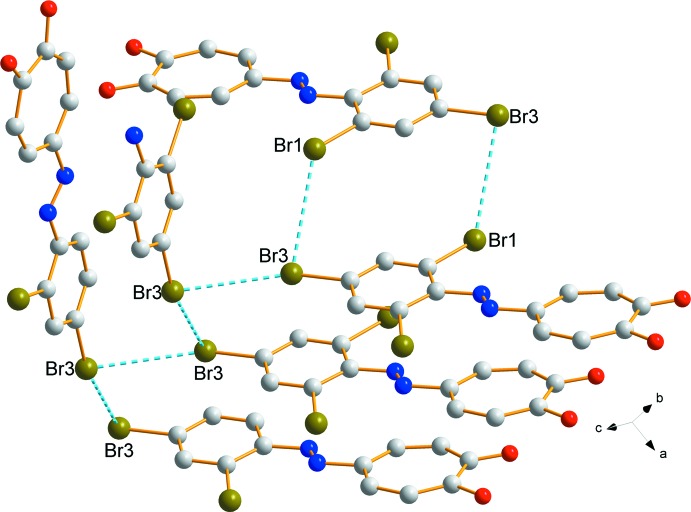
The Br⋯Br inter­actions of **I** illustrated with dashed bonds. Hydrogen atoms omitted for clarity.

As with tropolone (Shimanouchi & Sasada, 1973 ▸) along with the azotropolones, **I** forms centrosymmetric *homo*dimers through an O—H⋯O inter­action (Table 1 ▸, Fig. 2 ▸). Steyl & Roodt (2008 ▸) proposed a general range for Br⋯Br inter­actions, with distances between 3.6 and 4.6 Å with an inter­action angle of 40 to 180°. Two noteworthy Br⋯Br inter­actions are observed for compound **I**, firstly, a ring formed with the adjacent mol­ecule with a distance of 3.8246 (3) Å [Br1⋯Br3(−*x*, 1 − *y*, 1 − *z*)], and secondly, a chain linking the mol­ecules [Br3⋯Br3(−*x*, −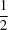 + *y*, 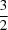 − *z*)] with a distance of 3.6841 (4) Å (Fig. 3 ▸). Although the triangle inter­action, which was observed by Steyl & Roodt, was present for **I**, the distances are rather long [3.6441 (4), 3.8246 (3) and 4.0113 (4) Å], but still fall into the range set.

The π–π inter­actions that were observed for 5-phenyl­azotropolone and 5-(4-eth­oxy­phenyl­azo)tropolone are not observed for **I**. Instead, a π→π^*^ charge-transfer (CT) inter­action between the aromatic phenyl and tropolone with the diazenyl group is seen (Fig. 2 ▸), with ring centroid to N=N midpoint distances of 3.3463 (3) and 3.3415 (3) Å, respectively. Additionally, a Br⋯π inter­action is found with a Br to benzene ring centroid distance of 3.4563 (3) Å.

As a result of the observed O—H⋯O, Br⋯Br, Br⋯π and π→π^*^ CT inter­actions, the assembly of **I** is seen as forming segregated stacks along the *b*-axis direction (Fig. 2 ▸).

## Hirshfeld Surface Analysis   

**Figure 4:**
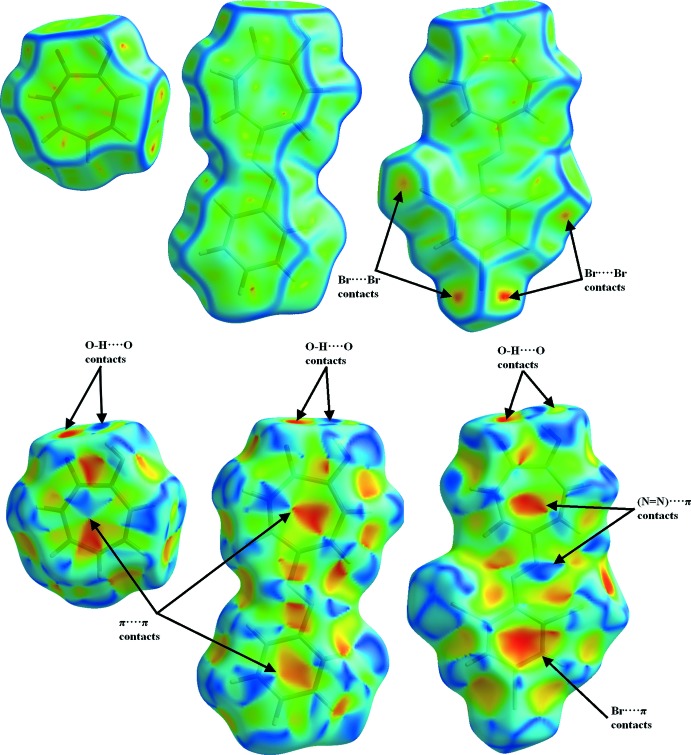
Hirshfeld surfaces for tropolone, 5-phenyl­tropolone and **I**, mapped with curvedness (top) and shape index (bottom).

Hirshfeld surface plots were generated for tropolone (CSD refcode: TROPOL10; Shimanouchi & Sasada, 1973 ▸), 5-phenyl­azotropolone (CSD refcode: YDIVYZ; Hill *et al.*, 2012 ▸) and **I** based on the crystallographic information file (CIF) using *CrystalExplorer*17.5 (Turner *et al.*, 2017 ▸), to explore and compare the location of atom-to-atom short contacts along with the qu­anti­tative ratios of these inter­actions. Unfortunatly, the Hirshfeld surfaces for 5-(4-eth­oxy­phenyl­azo)tropolone could not be generated as the co-ordinates were not inputted into the CSD. The curvedness plots of tropolone and 5-phenyl­tropolone show large regions of green. This is attributed to a relatively flat surface area (planar), whilst the blue regions illustrate areas of curvature (Fig. 4 ▸). For tropolone and 5-phenyl­tropolone, it can be seen that the mol­ecules are essentially planar, as mentioned previously, with 5-phenyl­tropolone having a dihedral angle (between the least-squares planes of the tropolone and phenyl moieties) of 1.57 (8)°. This is smaller than the corresponding angle found for **I** [5.07 (6)°], where the additional twist of the dihedral planes can clearly be seen in the curvedness plot, as there are additional blue regions which snake over the ‘planar’ surface of the mol­ecule. The curvedness plots of the compounds show flat surface areas, which is consistent with the planar packing arrangement that has been observed for both tropolone and 5-phenyl­azotropolone. It is inter­esting to note that in the curvedness plots, the Br⋯Br inter­actions are clearly visible as curved (red) regions and contribute 9% to the total surface area. These inter­actions are mirrored in the shape-index plots but are a little harder to observe.

On the shape-index surface plots for tropolone, 5-phenyl­azotropolone and **I** (Fig. 4 ▸), convex blue regions represent donor groups, whilst the red concave regions are the acceptor groups. The π–π inter­actions are generally indicated by adjacent red and blue triangles. These triangles are clearly observed for both tropolone (17.5% surface contribution) and 5-phenyl­tropolone (10.5% surface contribution), whereas this triangle formation was not found for **I**, further supporting the finding of no π–π inter­actions. As mentioned in the *Supra­molecular features* section, **I** is seen to have both Br⋯π (7.1% surface contribution) and CT (9.2% surface contribution) inter­actions (Fig. 2 ▸). This is further supported by the Hirshfeld shape-index surface illustrating the blue region of the donor azo group and the red accepting region of the tropolone moiety (Fig. 4 ▸).

## Synthesis and crystallization   

The reagents were commercially obtained and used without further purification.

Sodium nitrite (1.4 mmol) dissolved in water (1 cm^3^) was added dropwise to a solution containing 2,4,6-tri­bromo­aniline (1.6 mmol), hydro­chloric acid (2 cm^3^, conc.) and water (7 cm^3^). Upon cooling the resultant mixture to *ca* 277 K, it was added slowly to a solution of sodium hydroxide (1.8 mmol) and tropolone (1.6 mmol) in water (4 cm^3^), keeping the temperature below 278 K. The resulting solution was stirred for 30 min., filtered and air-dried. Crystals suitable for X-ray diffraction were obtained by recrystallization with CHCl_3_. Yield: 281 mg (38%), IR_ATR_: 3231, 1602, 1541, 1524, 1507, 1439, 1411, 1439, 1304, 1258, 1190, 1052, 872, 852, 799, 730, 705, 669, 620, 604, 546, 490 cm^−1^.

## Refinement   

Crystal data, data collection and structure refinement details are summarized in Table 2 ▸. All hydrogen atoms were positioned geometrically and refined using a riding model, with C—H = 0.95 Å, *U*_iso_(H) = 1.2*U*_eq_(C) for aromatic H atoms and with O—H = 0.84 Å, *U*_iso_(H)=1.5*U*_eq_(O) for the hy­droxy H atom.

## Supplementary Material

Crystal structure: contains datablock(s) global, I. DOI: 10.1107/S2056989018006151/fy2127sup1.cif

Structure factors: contains datablock(s) I. DOI: 10.1107/S2056989018006151/fy2127Isup2.hkl

Supporting information file. DOI: 10.1107/S2056989018006151/fy2127Isup3.cml

CCDC reference: 1838908

Additional supporting information:  crystallographic information; 3D view; checkCIF report

## Figures and Tables

**Table 1:** Hydrogen-bond geometry (Å, °)

*D*—H⋯*A*	*D*—H	H⋯*A*	*D*⋯*A*	*D*—H⋯*A*
C6—H6⋯N2	0.95	2.36	2.705 (3)	101
O2—H2⋯O1	0.84	2.1	2.591 (2)	117
C3—H3⋯O2^i^	0.95	2.54	3.241 (3)	131

Symmetry code: (i) 
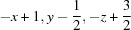
.

**Table 2:** Experimental details

Crystal data	
Chemical formula	C_13_H_7_Br_3_N_2_O_2_
*M*_r_	462.94
Crystal system, space group	Monoclinic, *P*2_1_/*c*
Temperature (K)	173
*a*, *b*, *c* (Å)	18.1742 (3), 4.7885 (1), 16.0308 (3)
β (°)	104.560 (1)
*V* (Å^3^)	1350.31 (4)
*Z*	4
Radiation type	Mo *K*α
μ (mm^−1^)	8.96
Crystal size (mm)	0.4 × 0.12 × 0.07

Data collection	
Diffractometer	Bruker APEXII CCD area detector
Absorption correction	Multi-scan (*SADABS*; Bruker, 2004 ▸)
*T*_min_, *T*_max_	0.124, 0.573
No. of measured, independent and observed [*I* > 2σ(*I*)] reflections	27599, 3258, 2744
*R*_int_	0.055
(sin θ/λ)_max_ (Å^−1^)	0.661

Refinement	
*R*[*F* ^2^ > 2σ(*F* ^2^)], *wR*(*F* ^2^), *S*	0.023, 0.051, 1.02
No. of reflections	3258
No. of parameters	182
H-atom treatment	H-atom parameters constrained
Δρ_max_, Δρ_min_ (e Å^−3^)	0.77, −0.68

Computer programs: *APEX2* (Bruker, 2005 ▸), *SAINT-Plus* and *XPREP* (Bruker, 2004 ▸), *SHELXS97* and *SHELXL97* (Sheldrick, 2008 ▸), *DIAMOND* (Brandenburg & Putz, 2005 ▸) and *WinGX* (Farrugia, 2012 ▸).

## supplementary crystallographic information

### Crystal data

**Table:**

C_13_H_7_Br_3_N_2_O_2_	*F*(000) = 880
*M**_r_* = 462.94	*D*_x_ = 2.277 Mg m^−^^3^
Monoclinic, *P*2_1_/*c*	Mo *K*α radiation, λ = 0.71073 Å
Hall symbol: -P 2ybc	Cell parameters from 8182 reflections
*a* = 18.1742 (3) Å	θ = 2.3–28.2°
*b* = 4.7885 (1) Å	µ = 8.96 mm^−^^1^
*c* = 16.0308 (3) Å	*T* = 173 K
β = 104.560 (1)°	Plate, red
*V* = 1350.31 (4) Å^3^	0.4 × 0.12 × 0.07 mm
*Z* = 4	

### Data collection

**Table:**

Bruker APEXII CCD area detector diffractometer	3258 independent reflections
Radiation source: fine-focus sealed tube	2744 reflections with *I* > 2σ(*I*)
Graphite monochromator	*R*_int_ = 0.055
Detector resolution: 512 pixels mm^-1^	θ_max_ = 28°, θ_min_ = 2.3°
φ and ω scans	*h* = −24→24
Absorption correction: multi-scan (SADABS; Bruker, 2004)	*k* = −6→6
*T*_min_ = 0.124, *T*_max_ = 0.573	*l* = −20→21
27599 measured reflections	

### Refinement

**Table:**

Refinement on *F*^2^	0 restraints
Least-squares matrix: full	H-atom parameters constrained
*R*[*F*^2^ > 2σ(*F*^2^)] = 0.023	*w* = 1/[σ^2^(*F*_o_^2^) + (0.0151*P*)^2^ + 1.5233*P*] where *P* = (*F*_o_^2^ + 2*F*_c_^2^)/3
*wR*(*F*^2^) = 0.051	(Δ/σ)_max_ = 0.001
*S* = 1.01	Δρ_max_ = 0.77 e Å^−^^3^
3258 reflections	Δρ_min_ = −0.68 e Å^−^^3^
182 parameters	

### Special details

**Table:**

Geometry. All esds (except the esd in the dihedral angle between two l.s. planes) are estimated using the full covariance matrix. The cell esds are taken into account individually in the estimation of esds in distances, angles and torsion angles; correlations between esds in cell parameters are only used when they are defined by crystal symmetry. An approximate (isotropic) treatment of cell esds is used for estimating esds involving l.s. planes.

### Fractional atomic coordinates and isotropic or equivalent isotropic displacement parameters (Å^2^)

**Table:**

	*x*	*y*	*z*	*U*_iso_*/*U*_eq_	
C1	0.41630 (13)	1.5974 (5)	0.51392 (14)	0.0173 (5)	
C2	0.45286 (13)	1.6351 (5)	0.60559 (14)	0.0171 (4)	
C3	0.43788 (13)	1.5012 (5)	0.67536 (15)	0.0189 (5)	
H3	0.4684	1.5592	0.7298	0.023*	
C4	0.38490 (13)	1.2934 (5)	0.67894 (14)	0.0191 (5)	
H4	0.3844	1.2338	0.7353	0.023*	
C5	0.33337 (12)	1.1622 (5)	0.61327 (14)	0.0163 (4)	
C6	0.32247 (13)	1.2070 (5)	0.52358 (14)	0.0181 (5)	
H6	0.285	1.0929	0.4873	0.022*	
C7	0.35821 (13)	1.3907 (5)	0.48232 (15)	0.0190 (5)	
H7	0.3419	1.3811	0.4213	0.023*	
C11	0.20015 (13)	0.6200 (5)	0.62034 (14)	0.0171 (4)	
C12	0.14250 (13)	0.4963 (5)	0.55595 (14)	0.0184 (5)	
C13	0.09308 (13)	0.2960 (5)	0.57313 (15)	0.0208 (5)	
H13	0.0542	0.2182	0.5282	0.025*	
C14	0.10233 (13)	0.2135 (5)	0.65781 (16)	0.0213 (5)	
C15	0.15835 (14)	0.3243 (5)	0.72361 (15)	0.0208 (5)	
H15	0.1639	0.263	0.7812	0.025*	
C16	0.20691 (13)	0.5268 (5)	0.70536 (14)	0.0186 (5)	
N1	0.28798 (11)	0.9647 (4)	0.64541 (13)	0.0221 (4)	
N2	0.24557 (11)	0.8171 (4)	0.58994 (12)	0.0200 (4)	
O1	0.43793 (10)	1.7538 (4)	0.46231 (10)	0.0230 (4)	
O2	0.50700 (10)	1.8299 (3)	0.62292 (11)	0.0225 (4)	
H2	0.511	1.9037	0.5767	0.034*	
Br1	0.128145 (15)	0.60750 (6)	0.439523 (15)	0.02966 (7)	
Br2	0.282839 (16)	0.65030 (6)	0.802286 (15)	0.03009 (8)	
Br3	0.036554 (14)	−0.06019 (5)	0.684700 (18)	0.02738 (7)	

### Atomic displacement parameters (Å^2^)

**Table:**

	*U*^11^	*U*^22^	*U*^33^	*U*^12^	*U*^13^	*U*^23^
C1	0.0184 (11)	0.0153 (11)	0.0192 (11)	0.0027 (9)	0.0069 (9)	0.0010 (9)
C2	0.0160 (11)	0.0147 (10)	0.0201 (11)	0.0005 (8)	0.0038 (9)	−0.0016 (9)
C3	0.0193 (11)	0.0198 (11)	0.0164 (11)	−0.0005 (9)	0.0021 (9)	0.0002 (9)
C4	0.0199 (12)	0.0211 (12)	0.0155 (11)	0.0000 (9)	0.0029 (9)	0.0042 (9)
C5	0.0150 (11)	0.0146 (10)	0.0206 (11)	0.0007 (9)	0.0068 (9)	0.0025 (9)
C6	0.0179 (11)	0.0156 (11)	0.0195 (11)	−0.0001 (9)	0.0022 (9)	−0.0011 (9)
C7	0.0208 (12)	0.0202 (12)	0.0152 (11)	0.0008 (9)	0.0030 (9)	0.0006 (9)
C11	0.0164 (11)	0.0142 (11)	0.0217 (11)	0.0004 (8)	0.0064 (9)	0.0004 (9)
C12	0.0178 (11)	0.0190 (11)	0.0191 (11)	0.0018 (9)	0.0060 (9)	0.0013 (9)
C13	0.0183 (12)	0.0177 (11)	0.0266 (12)	−0.0010 (9)	0.0060 (10)	−0.0033 (9)
C14	0.0200 (12)	0.0135 (11)	0.0347 (13)	0.0007 (9)	0.0146 (10)	0.0009 (10)
C15	0.0248 (12)	0.0179 (11)	0.0221 (12)	0.0019 (10)	0.0102 (10)	0.0021 (9)
C16	0.0203 (12)	0.0165 (11)	0.0195 (11)	0.0000 (9)	0.0058 (9)	−0.0020 (9)
N1	0.0217 (10)	0.0220 (10)	0.0224 (10)	−0.0043 (8)	0.0055 (8)	0.0043 (8)
N2	0.0185 (10)	0.0199 (10)	0.0217 (10)	−0.0047 (8)	0.0051 (8)	0.0012 (8)
O1	0.0287 (9)	0.0202 (8)	0.0207 (8)	−0.0039 (7)	0.0076 (7)	0.0027 (7)
O2	0.0253 (9)	0.0214 (8)	0.0213 (8)	−0.0088 (7)	0.0070 (7)	0.0012 (7)
Br1	0.02972 (14)	0.03815 (15)	0.01879 (12)	−0.00894 (11)	0.00180 (10)	0.00365 (10)
Br2	0.04013 (16)	0.03091 (14)	0.01657 (12)	−0.01094 (11)	0.00216 (10)	0.00090 (10)
Br3	0.02564 (13)	0.01662 (12)	0.04556 (16)	−0.00247 (10)	0.01955 (11)	0.00277 (11)

### Geometric parameters (Å, º)

**Table:**

C1—O1	1.250 (3)	C11—C12	1.404 (3)
C1—C7	1.443 (3)	C11—C16	1.410 (3)
C1—C2	1.464 (3)	C11—N2	1.419 (3)
C2—O2	1.333 (3)	C12—C13	1.388 (3)
C2—C3	1.375 (3)	C12—Br1	1.895 (2)
C3—C4	1.396 (3)	C13—C14	1.383 (3)
C3—H3	0.95	C13—H13	0.95
C4—C5	1.373 (3)	C14—C15	1.375 (3)
C4—H4	0.95	C14—Br3	1.895 (2)
C5—C6	1.417 (3)	C15—C16	1.391 (3)
C5—N1	1.433 (3)	C15—H15	0.95
C6—C7	1.360 (3)	C16—Br2	1.895 (2)
C6—H6	0.95	N1—N2	1.241 (3)
C7—H7	0.95	O2—H2	0.84
			
O1—C1—C7	120.2 (2)	C12—C11—C16	116.4 (2)
O1—C1—C2	116.8 (2)	C12—C11—N2	114.79 (19)
C7—C1—C2	123.0 (2)	C16—C11—N2	128.8 (2)
O2—C2—C3	116.3 (2)	C13—C12—C11	123.1 (2)
O2—C2—C1	114.9 (2)	C13—C12—Br1	117.00 (17)
C3—C2—C1	128.8 (2)	C11—C12—Br1	119.88 (17)
C2—C3—C4	130.2 (2)	C14—C13—C12	117.9 (2)
C2—C3—H3	114.9	C14—C13—H13	121.1
C4—C3—H3	114.9	C12—C13—H13	121.1
C5—C4—C3	129.7 (2)	C15—C14—C13	121.8 (2)
C5—C4—H4	115.1	C15—C14—Br3	118.70 (18)
C3—C4—H4	115.1	C13—C14—Br3	119.53 (18)
C4—C5—C6	127.1 (2)	C14—C15—C16	119.6 (2)
C4—C5—N1	111.70 (19)	C14—C15—H15	120.2
C6—C5—N1	121.2 (2)	C16—C15—H15	120.2
C7—C6—C5	129.0 (2)	C15—C16—C11	121.2 (2)
C7—C6—H6	115.5	C15—C16—Br2	114.52 (17)
C5—C6—H6	115.5	C11—C16—Br2	124.22 (17)
C6—C7—C1	132.0 (2)	N2—N1—C5	115.36 (19)
C6—C7—H7	114	N1—N2—C11	116.30 (19)
C1—C7—H7	114	C2—O2—H2	109.5
			
O1—C1—C2—O2	−1.7 (3)	C11—C12—C13—C14	−0.9 (4)
C7—C1—C2—O2	178.0 (2)	Br1—C12—C13—C14	−179.57 (17)
O1—C1—C2—C3	177.0 (2)	C12—C13—C14—C15	0.0 (4)
C7—C1—C2—C3	−3.4 (4)	C12—C13—C14—Br3	−179.80 (17)
O2—C2—C3—C4	179.1 (2)	C13—C14—C15—C16	0.6 (4)
C1—C2—C3—C4	0.5 (4)	Br3—C14—C15—C16	−179.58 (17)
C2—C3—C4—C5	0.9 (5)	C14—C15—C16—C11	−0.4 (4)
C3—C4—C5—C6	0.9 (4)	C14—C15—C16—Br2	−178.21 (18)
C3—C4—C5—N1	−178.5 (2)	C12—C11—C16—C15	−0.4 (3)
C4—C5—C6—C7	−1.8 (4)	N2—C11—C16—C15	−178.1 (2)
N1—C5—C6—C7	177.6 (2)	C12—C11—C16—Br2	177.20 (17)
C5—C6—C7—C1	−1.0 (4)	N2—C11—C16—Br2	−0.5 (4)
O1—C1—C7—C6	−176.5 (2)	C4—C5—N1—N2	−172.8 (2)
C2—C1—C7—C6	3.9 (4)	C6—C5—N1—N2	7.7 (3)
C16—C11—C12—C13	1.1 (3)	C5—N1—N2—C11	179.97 (19)
N2—C11—C12—C13	179.1 (2)	C12—C11—N2—N1	167.9 (2)
C16—C11—C12—Br1	179.71 (16)	C16—C11—N2—N1	−14.3 (4)
N2—C11—C12—Br1	−2.3 (3)		

### Hydrogen-bond geometry (Å, º)

**Table:**

*D*—H···*A*	*D*—H	H···*A*	*D*···*A*	*D*—H···*A*
C6—H6···N2	0.95	2.36	2.705 (3)	101
O2—H2···O1	0.84	2.1	2.591 (2)	117
C3—H3···O2^i^	0.95	2.54	3.241 (3)	131

Symmetry code: (i) −*x*+1, *y*−1/2, −*z*+3/2.
